# A safe therapeutic strategy for giant pedunculated colorectal polyps with thick stalks

**DOI:** 10.1055/a-2191-2421

**Published:** 2023-11-20

**Authors:** Takehide Fukuchi, Shigeru Iwase, Shinpei Kondo, Shin Maeda

**Affiliations:** 1 36993 Department of Gastroenterology, Fujisawa City Hospital, Fujisawa, Japan; 2 26438 Gastroenterology Division, Yokohama City University School of Medicine Graduate School of Medicine, Yokohama, Japan

Endoscopic resection of giant pedunculated colorectal polyps (PCPs) with large heads and thick stalks can be technically difficult with conventional snare polypectomy and involves the risk of clinically significant bleeding 1 . The European Society of Gastrointestinal Endoscopy guideline recommends pretreatment of the stalk with a dilute epinephrine injection and mechanical hemostasis (prophylactic clip and endoloop application) for PCPs 2 3 . Endoscopic submucosal dissection (ESD) can control hemostasis 1 4 5 . However, some cases comprising large tumor heads are associated with challenging operability and poor visibility, so achieving hemostasis can be difficult. We have developed a strategy to manage bleeding during colorectal ESD of PCPs with thick stalks ( Video 1 ).

A safe therapeutic strategy is used to resect a giant pedunculated colorectal polyp with a thick stalk.Video 1

A 61-year-old man underwent colonoscopy for intermittent hematochezia for 3 months; the results revealed a giant PCP in the sigmoid colon that was almost completely blocking the lumen ( Fig. 1 ). Computed tomography showed the inflow of a thick artery into the tumor. There was no clear sign of submucosal invasion. We created an incision from the anal side of the tumor using an ESD knife (MICRO-TECH, Nanjing, China). After exposing the anal side, a novel clip-band device (SureClip traction band; MICRO-TECH) was attached to the stalk, and the other end of the elastic ring was hooked onto the SureClip and clipped to the anal side of the intestinal tract ( Fig. 2 ). Protruding lesions often include fibrosis and muscularis traction. The artery within the fibrosis therefore had to be carefully dissected to avoid damaging it ( Fig. 3 ). Finally, we applied two clips to clamp the central tissue containing the thick vessels. As a result of this, only minimal bleeding occurred when cutting between the clips was performed. The lesion was removed using curative R0 resection, and intraoperative bleeding was completely controlled.

**Fig. 1:**
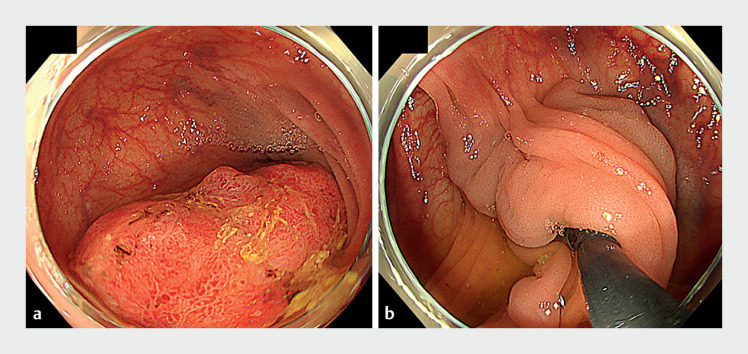
Endoscopic images of a giant pedunculated colorectal polyp at the sigmoid colon showing: **a** the head of the tumor; **b** the stalk of the tumor.

**Fig. 2:**
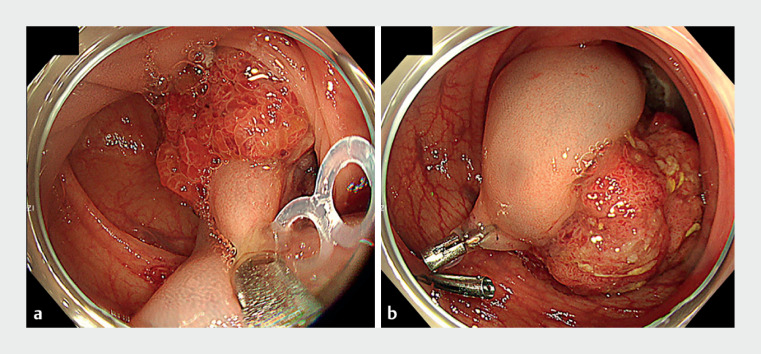
Endoscopic images of an effective technique using a traction device for a giant pedunculated colorectal polyp with a thick stalk showing: **a** the traction band clip attached to the stalk; **b** the tumor fixed with clips.

**Fig. 3:**
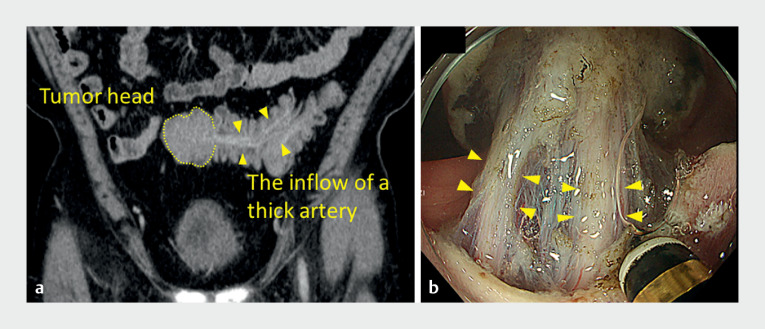
Thick vessels are present inside the stalks of giant pedunculated colorectal polyps as shown on: **a** computed tomography; **b** endoscopy.

Planned treatment strategies are essential in the prevention of intraoperative complications associated with giant PCPs with thick stalks.

Endoscopy_UCTN_Code_TTT_1AQ_2AD

## Notes

Endoscopy E-Videos https://eref.thieme.de/e-videos
*E-Videos* is an open access online section of the journal *Endoscopy* , reporting on interesting cases and new techniques in gastroenterological endoscopy. All papers include a high-quality video and are published with a Creative Commons CC-BY license. Endoscopy E-Videos qualify for HINARI discounts and waivers and eligibility is automatically checked during the submission process. We grant 100% waivers to articles whose corresponding authors are based in Group A countries and 50% waivers to those who are based in Group B countries as classified by Research4Life (see: https://www.research4life.org/access/eligibility/ ). This section has its own submission website at https://mc.manuscriptcentral.com/e-videos .
